# Physician professionalism: definition from a generation perspective

**DOI:** 10.5116/ijme.5ba0.a584

**Published:** 2018-09-28

**Authors:** Wirachin Hoonpongsimanont, Preet K. Sahota, Yanjun Chen, Mayuri Patel, Tanawat Tarapan, Deena Bengiamin, Krongkarn Sutham, Intanon Imsuwan, Ar-aishah Dadeh, Tanyaporn Nakornchai, Khuansiri Narajeenron

**Affiliations:** 1 Department of Emergency Medicine, University of California, Irvine, Orange, CA, USA; 2 Institute for Clinical and Translational Sciences, University of California, Irvine, Irvine, CA, USA; 3 Department of Emergency Medicine, University and King Chulalongkorn Memorial Hospital, the Thai Red Cross Society, Bangkok, Thailand; 4 Department of Emergency Medicine, University of California, San Francisco-Fresno, Fresno, CA, USA; 5 Department of Emergency Medicine, Chiang Mai University, Chiang Mai, Thailand; 6 Department of Emergency Medicine, Thammasat University, Pathumthani, Thailand; 7 Department of Emergency Medicine, Prince of Songkla University, Songkla, Thailand; 8 Department of Emergency Medicine, Siriraj Hospital, Mahidol University, Bangkok, Thailand

**Keywords:** Professionalism, emergency physician, generation, perception, quantitative cultural consensus

## Abstract

**Objectives:**

The primary objective of this study was to determine whether consensuses on the definition of emergency physician professionalism exist within and among four different generations. Our secondary objective was to describe the most important characteristic related to emergency physician professionalism that each generation values.

**Methods:**

We performed a cross-sectional survey study, using a card-sorting technique, at the emergency departments of two university-based medical centers in the United States. The study was conducted with 288 participants from February to November 2017. Participants included adult emergency department patients, emergency medicine supervising physicians, emergency medicine residents, emergency department nurses, and fourth- and second-year medical students who independently ranked 39 cards that represent qualities related to emergency physician professionalism. We used descriptive statistics, quantitative cultural consensuses and Spearman’s correlation coefficients to analyze the data.

**Results:**

We found cultural consensuses on emergency physician professionalism in Millennials and Generation X overall, with respect for patients named the most important quality (eigenratio 5.94, negative competency 0%; eigenratio 3.87, negative competency 1.64%, respectively). There were consensuses on emergency physician professionalism in healthcare providers throughout all generations, but no consensuses were found across generations in the patient groups.

**Conclusions:**

While younger generations and healthcare providers had consensuses on emergency physician professionalism, we found that patients had no consensuses on this matter. Medical professionalism curricula should be designed with an understanding of each generation’s values concerning professionalism. Future studies using qualitative methods across specialties, to assess definitions of medical professionalism in each generation, should be pursued.

## Introduction

The rise of public dissatisfaction with physicians in general and the downward trend in positive perceptions of physicians’ altruism create strained physician-patient relationships that in turn result in malpractice lawsuits.^1^ Altruism is just one facet of professionalism, a skillset that has recently become a cornerstone of medicine and medical education.^2^ A better understanding of professionalism may help reduce physician self-interest and corporate influence within medicine while improving patient trust, patient satisfaction and the patient-physician relationship as a whole.^3^^,^^4^ Numerous efforts have been undertaken to improve the teaching of medical professionalism. The Accreditation Council for Graduate Medical Education named professionalism one of six core competencies, thus mandating all residency training programs to teach and evaluate professionalism.^5^

Yet no single, universal definition of physician professionalism exists, instead, healthcare experts and educators have described professionalism using a number of behaviors and characteristics that include fidelity to patient trust, accountability, respect, compassion, integrity, sound ethics, altruism and truthfulness.^6^^,^^7^ Previous studies identified concrete examples of physician behaviors that patients regard as professional, such as formally greeting patients with a handshake and taking notes while speaking to the patient.^8^ There have been mixed results concerning the importance of wearing a white coat with regard to physician professionalism.^8^^,^^9^ Individual backgrounds, cultures, socioeconomic status and age or generation affect how each person perceives medical professionalism. To date, emergency physician (EP) professionalism definitions are yet to be established.

The importance of physician professionalism is even more prominent in the unique setting of the emergency department (ED). The combination of vulnerable and complex patients, longer waiting times and ED crowding obliges EPs to perform at a higher standard of professionalism.^10^

The ED setting also brings patients and medical practitioners from various cultures and generations together. These include Millennials (ages 17-36), Generation X (ages 37-52), Baby Boomers (ages 53-71) and the Silent Generation (ages 72-89).^11^^,^^12^^,^^13^ Although individuals belonging to a particular generation are far from identical, they do tend to share common historical events and similar life experiences that lead to similar behaviors, values and perceptions.

The Silent Generation consists of individuals born from 1928 to 1945. This cohort is generally perceived as tending to work hard, not speak out, and conform to societal expectations. They were born during World War II and the Great Depression and reached adulthood in the comparatively prosperous 1950s and 1960s.^12^

Baby Boomers are defined as the demographic cohort born from 1946 through 1964. Perhaps because these individuals were raised in a time of great social change and upheaval, they lean toward moderate and conservative viewpoints.^13^ Baby Boomers are commonly viewed as competitive, dedicated and hardworking. They value individuality and loyalty to their jobs.^14^

Generation X includes those born from 1965 to 1980, an era characterized by increased divorce rates. These individuals were often raised in single-parent homes and thus had less supervision and bonding time with their parents who were generally away working. This group was characterized as pessimistic and unsatisfied during their adolescent and young adult years.^15^ Now in adulthood, they are described as a whole as being informal, independent, fun-loving, active and happy, and with an appreciation for work-life balance.^14^^,^^16^

Millennials or Generation Y are those born from 1981 to 2000. Growing up, this group often had supportive “helicopter” parents who were closely, perhaps excessively, concerned with their children’s success and well-being. This generation has been described as confident, socially sensitive, conventional, ambitious and achieving.^14^^,^^17^

The values and characteristics associated with each generation of individuals may influence how they define and perceive medical professionalism. Literature that examines the link between generations and perceptions of medical professionalism is limited. Previous studies have explored how age can affect an individual’s likelihood to consider certain behaviors as professional or unprofessional in healthcare settings.^18^

Our study aims to identify the gap in the definition of EP professionalism (EPP) within and among generations. The primary objective of this study was to discover whether consensuses on the definition of EPP exist within and among the four different generations. Our secondary objective was to describe the most important characteristic related to EPP that each generation values.

## Methods

### Study design and setting

We conducted a cross-sectional survey study, using a card-sorting technique, at the EDs of two university-based medical centers in the United States (US). The study was conducted from February to November 2017. We included adults 18 years and older who were participants in the healthcare system, including ED patients, emergency medicine (EM) supervising physicians, EM residents, ED nurses and fourth- and second-year medical students. Only English-speaking participants were approached. We excluded participants who had alterations of consciousness, severe pain, HIV/AIDS and cancer, and those who were pregnant, under psychiatric hold, under police custody and/or handicapped. We also excluded participants who were unable to provide verbal consent. The institutional review boards of the University of California, both at the Irvine campus and the San Francisco at Fresno campus, approved the study under the Exempt Category 2, which entails the following: “interactions involving educational tests, survey, interview procedures, or observation of public behavior (including visual or auditory recording).”^19^

We enrolled a total of 288 participants (Table 1). The majority (70.49%) were Millennials, and predominantly female. Less than half of enrolled patients were Millennials, whereas more than three-fourths of healthcare providers were Millennials. Overall, we enrolled more female participants in the patient and healthcare provider combined group.

### Study procedure and tool development

We reviewed available, published literature to identify 13 elements that potentially represent EPP.^20^^-^^29^ For each element, we created three cards, using face validity from expert opinions, with each card describing a unique quality or behavior associated with the given element. We ensured the accuracy and uniformity of wording/interpretation of each card by testing the cards with EM supervising physicians, EM residents, medical students and patients. After the revisions were made, we piloted the instrument with 25 ED patients. After several iterations, we achieved a deck of 39 cards, with each card describing a quality or behavior related to EPP (Appendix).

**Table 1:** Demographics of the participant by generation (N=288)

Demographic Variable	Millennials		Generation X		Baby Boomers/Silent Generation		Overall
	N	%	N	%	N	%	N
Overall (Patients and Healthcare Providers)							
Total	203	70.49	61	21.18	24	8.33	288
Female	104	70.27	31	20.95	13	8.78	148
Male	99	70.71	30	21.43	11	7.86	140
Patients							
Total	24	46.15	14	26.92	14	26.92	52
Female	16	45.71	11	31.43	8	22.86	35
Male	8	47.06	3	17.65	6	35.29	17
Healthcare Providers							
Total	179	75.85	47	19.92	10	4.24	236
Female	88	77.88	20	17.70	5	4.42	113
Male	91	73.98	27	21.95	5	4.07	123

After participants verbally consented to participate, research associates asked them to independently rank all 39 cards from the most important behavior to the least important behavior contributing to EPP. They did this in a private room, without interference from the research team. Participants also completed a demographics survey, which consisted of gender, age, race, and questions about their religious affiliation. Study participants were also given the opportunity to describe an unprofessional encounter they may have experienced with a physician. We recorded all data in the secure Research Electronic Data Capture software, which is designed to store and manage online surveys and databases.

### Data analysis

To obtain a 0.5 competency (0.25 agreement based on averaged Pearson correlation coefficients) and 95% validity, we needed at least 28 participants in each cohort.30 Descriptive statistics for participant demographics were provided. We used the quantitative cultural consensus method to examine the existence of a shared cultural model and to determine the aggregated rankings within each cohort.^30^ The cultural consensus method is a statistical model that evaluates the degree of agreement within groups and estimates the ‘culturally shared’ belief where the answer was previously unknown. We used factor analysis of respondents, using minimum-residual algorithm (no rotation), to assess the degree of agreement within one cohort and obtain the individuallevel competency scores. The method assumes that there is only a single factor solution. The ratio between the first and second eigenratios (E) indicate whether there is only a single shared dimension in the data. E 3 to 1 or greater indicates a shared cultural idea within the cohort. Competency levels for each individual were estimated as the first-factor loading from the factor analysis. A higher average competency score indicates a higher within-cohort consensus level. Negative competency (NC) scores indicate the presence of more than one sub-cultural group. Less than 5% NC scores were considered acceptable in each cohort. If the above two criteria were met, aggregated rankings of the cohort were estimated as the first set of factor scores. Finally, Spearman’s correlation coefficient was used to examine the aggregated ranking between cohorts. We used SAS version 9.4 to conduct all data analyses (SAS Institute Inc 2013. SAS/ACCESS® 9.4 Cary, NC).

**Table 2:** Cultural consensus model on emergency physician professionalism for each generational cohort

Generation	N	E	Competency		NC%
			Mean	SD	
Overall					
Millennials	203	5.94	0.59	0.16	0.00
Generation X	61	3.87	0.53	0.19	1.64
Baby Boomers/Silent Generation	24	2.34	0.44	0.27	12.50
Healthcare Providers					
Millennials	179	6.72	0.62	0.13	0.00
Generation X	47	4.23	0.57	0.17	0.00
Baby Boomers/Silent Generation	10	3.39	0.54	0.23	0.00
Patients					
Millennials	24	2.77	0.45	0.27	4.17
Generation X	14	2.19	0.46	0.22	0.00
Baby Boomers/Silent Generation	14	3.01	0.44	0.28	7.14

Key: E: eigenratios; NC: negative competency; Note: Competency and negative competency values for each cohort per generation; eigenratios depict shared dimensions.

## Results

The data showed consensuses on EPP in Millennials and Generation X when analyzing both healthcare providers and patients as one group (E 5.94, NC 0%; E 3.87, NC 1.64%, respectively), see Table 2. The Millennials showed higher consensus (average competency score = 0.59), followed by Generation X (average competency score = 0.53). Combining patients and healthcare providers in one group, Millennials and Generation X both ranked respect for patients as the most important quality. Being trustworthy and dependable and compassionate patient care were ranked in the top five for both generations. In contrast, complete medical records on time, adhere to doctor’s religious and moral values, appropriate grooming, professional attire and wearing a white coat were ranked as the five least important qualities for EPP in all three cohorts (Table 3).

**Table 3:** Aggregated rankings of professional qualities in overall and healthcare provider groups

Professional qualities	Overall		Healthcare Providers		
	M	X	M	X	B
Unselfishness	22	26	22	28	12
Choose patient's interest over physician's interest	14	19	14	17	5
Desire to help others	4	9	5	12	6.5
Being patient’s advocate	5	7	4	7	2
Being responsible to colleagues	20	23	20	19	9
Being responsible to society and institution	26	27	26	26	26
Self-motivated to practice excellence by being a lifelong learner	18	21	16	18	10
Teach other medical personnel	32	28	29	27	15
Promote research to create new knowledge	33	30	33	30	23
Being transparent and truthful	7	3	7	2	3
Maintain the honor of the medical profession	23	20	25	23	25
Being trustworthy and dependable	2	2	2	1	1
Respect for patients	1	1	1	3	11
Politeness	21	17	21	22	21
Respect for co-workers	13	12	13	10	18
Communicate clearly to patients	9	6	9	6	8
Having proper conversation with co-worker	28	25	28	20	27
Listen and respond to patient's concern	6	8	6	9	4
Being a good team player	10	11	10	8	14
Good leadership	16	13.5	15	13	28.5
Self-control	24	16	24	16	31
Compassionate patient care	3	4	3	5	6.5
Share the feeling of patient's suffering	19	22	19	25	20
Kindness	11	10	11	11	13
Avoid any conflict of interest	29	31	30	33	30
Respect patient confidentiality and privacy	15	18	17	21	19
Adhere to doctor's religious and moral values	36	38	35	38	24
Holistic approach to patients	31	32	31	32	28.5
Excellent knowledge and procedural skills	8	5	8	4	17
Able to treat patients in various situations with limit resources	17	13.5	18	14	22
Responsive to feedback	25	24	23	24	32
Try new behaviors to promote patient care	34	34	34	34	35
Situational awareness	12	15	12	15	16
Wearing a white coat	39	39	39	39	39
Professional attire	38	37	38	36	37
Appropriate grooming	37	35	37	35	36
Complete medical records on time	35	36	36	37	38
Document charts accurately	30	33	32	31	33
Gather data efficiently with limited time and resources	27	29	27	29	34

Key: M: Millennials; X: Generation X; B: Baby Boomers/Silent Generation

Numbers represent rankings for each professional quality by each cohort; one is the most important quality and thirty-nine is the least important quality. Bolded numbers indicate values of the groups that have a cultural consensus

**Table 4:** Correlations of aggregated ranking by generation

Variables	Millennials	Generation X	Baby Boomers/ Silent Generation
Overall			
Millennials	1.000		
Generation X	0.998	1.000	
Healthcare Providers			
Millennials	1.000		
Generation X	0.997	1.000	
Baby Boomers/ Silent Generation	0.976	0.969	1.000

Note: Spearman’s correlation coefficients illustrate aggregated ranking between cohorts by generation. Bolded numbers indicate values of the groups that have a cultural consensus.

These two generations (Millennials and Generation X for the patient and healthcare provider combined group) showed a high correlation in aggregated ranking (0.998), see Table 4.  In the Baby Boomers and Silent Generation group, however, no consensus was appreciable (E 2.34). When analysing healthcare providers and patients separately, we found significant consensuses on EPP in healthcare providers throughout all generations (Millennial: E 6.72, Generation X: E 4.23, Baby Boomers/Silent Generation: E 3.39; All NC: 0%). Millennials expressed the highest level of consensus, with a mean competency score of 0.62, while the average competency scores of Generation X and Baby Boomers/Silent Generation were slightly lower at 0.57 and 0.54, respectively (Table 2). In addition, correlations of aggregated rankings are high among the three cohorts (greater than 0.95), see Table 4. In the healthcare provider group, Millennials ranked respect for patients, whereas Generation X and Baby Boomers/Silent Generation ranked being trustworthy and dependable, as the most important quality for EPP. The five least important qualities were similar in Millennials and Generation X, as compared to the combined patient and healthcare providers group. However, the Baby Boomers/Silent Generation generations considered adhere to doctor's religious and moral values more important in contrast to the other two health care provider generation groups (Table 3).  In the patient groups, no consensuses on EPP were found within any generation (Table 2).

## Discussion

In an attempt to define EPP, we were unable to demonstrate consensuses on the definition of EPP across all participants and generations. However, we were able to establish a greater understanding of the perceptions concerning EPP according to age and generations. Millennials tend to agree on what they value as professionalism whereas the other generations are still in controversy. If we only focus on which quality each generation considered to be the most important for EPP, Millennial providers valued respect whereas other generations valued trust and dependability. Given that today’s healthcare providers come from all four generations, these generational differences can give rise to conflicts in the workplace.^31^

In terms of patient care, the majority of healthcare providers in Generation X felt that being trustworthy and dependable are critical components of professionalism. While Generation X providers form their practice style to be trustworthy and dependable, patients from older and younger generations, non-consensually, seek other qualities, i.e., compassion, kindness and respect in their care. While these are all important components of professionalism, an unawareness of the differences in perceptions of EPP among generations may result in misinterpretations of patients and job dissatisfaction in providers. Our findings emphasize that generational differences in the perceptions of EPP exist and health care providers should be cognizant about these differences when treating patients. Understanding the patient’s perspective will aid healthcare providers in building rapport and trust, eventually leading to more positive clinical outcomes.^32^

Interestingly, we found that Millennials and Generation X share the same important value, respect for patients, although these two generations view respect differently. Older populations may not appreciate the equal respect and wish to be treated with more respect compared to others. In contrast, Millennials believe that respect must be earned and not simply given because of seniority status.^33^

Previous studies have indicated differences in the perception of professionalism in patients of different age groups.^34^ One study examined the perception of professionalism in medical students, residents and supervising physicians and found that each group ranked and valued professional behaviors differently, suggesting a generational influence on the perception of professionalism.^35^ Literature examining how patients rank different behaviors related to physician professionalism is limited. We did not find consensuses on EPP among patients, regardless of their generations. However, we recognized a general trend in which patients from the Millennial, Generation X and Baby Boomer/Silent Generation groups ranked respect for patient, compassionate patient care and kindness, respectively, as the most important values for EPP (although these were not statistically supported consensuses). This serves as a reminder that each individual is unique and should be treated, as deemed appropriate, differently. Patients’ personal experiences shape and will continue to change their perspectives of physician professionalism.

Results from Wiggins and colleagues demonstrated that patients consistently ranked behaviors related to communications skills as very important, and actions such as hand-washing as less important. Behaviors related to physician demeanor, such as putting the patient at ease, were ranked as intermediate.^36^ All generations in our study ranked physician appearance, including wearing a white coat, professional attire and appropriate grooming, as the least important qualities of EPP. This finding contradicts what has been taught and is expected from medical students during their training years. Perhaps less emphasis on students’ appearance and more focus on their ability to show respect and humanism will be the future of professionalism-centred curricula.

### Limitations

Our study has specific limitations that must be addressed. First, we have small sample sizes in certain participant groups, particularly in the Baby Boomer/Silent Generation groups. These smaller sample sizes may be a reason for non-consensuses in these groups. Larger sample sizes are needed to determine significant consensuses in certain subgroups. Secondly, asking a participant to rank 39 cards could possibly be overwhelming, although an expert in the card-sorting technique suggests that participant’s attention drops after ranking more than 40 cards.^37^ Finally, we would need data from more than one region to represent the US as a whole, and for the results to be generalizable and well-represented.

## Conclusions

Our study demonstrated that differences in perceptions of EPP do in fact exist among generations in this sample. More often, we see that health care providers share the same values and beliefs, but patients do not have uniform consensuses on the qualities of physician professionalism. Healthcare providers should be aware of this lack of consensus and customize their healthcare delivery to each individual patient. Medical professionalism curricula and evaluations should be designed with an understanding of generational differences in values of professionalism. Future studies using qualitative methods to assess the definition of medical professionalism, in each generation, should be pursued. Professionalism studies across various specialties are also warranted.

### Acknowledgments

The authors would like to thank Dr Bharath Chakravarthy and Dr Craig Anderson for their contribution to the research methodology for this project.

### Conflict of Interest

The authors declare that they have no conflict of interest.

## Supplementary material

Supplementary file 1 Appendix. Summary of the 39 cards used for the card-sorting task
