# Supplementary material for: Physician professionalism: definition from a generation perspective
Source: Int J Med Educ. 2018 Sep 28;9:246–52. doi: 10.5116/ijme.5ba0.a584 (PMC6387766; doi:10.5116/ijme.5ba0.a584)
Supplement: Supplementary file 1 — Appendix. Summary of the 39 cards used for the card-sorting task [file ijme-9-246-S1.pdf]

Appendix

Summary of the 39 cards used for the card-sorting task

Element	Card 1	Card 2	Card 3
Altruism	Unselfishness	Choose patient's interest over physician's interest	Desire to help others
Accountability	Being patient's advocate i.e. coordinate care efficiently, diligent regarding patient safety, consider the financial burden to the patient	Being responsible to colleagues i.e., consider co-worker's safety, defend colleague's slander misunderstanding	Being responsible to society and institution i.e., promote societal wellness, comply with institution's policies, efficient use of healthcare resources
Excellence	Self-motivated to practice excellence by being a lifelong learner	Teach other medical personnel	Promote research to create new knowledge
Honor and Integrity	Being transparent and truthful	Maintain the honor of the medical profession	Being trustworthy and dependable
Respect	Respect for patients i.e., consider cultural, individual, and role differences, shared decision making	Politeness i.e., greeting and self-introduction	Respect for co-workers
Communication Skills	Communicate clearly to patients i.e., use simple language	Have proper conversations with co-workers	Listen and respond to patient's concerns
Teamwork and Leadership	Being a good team player	Good leadership	Self-control i.e., avoiding emotional outbursts in a stressful situation
Humanism	Compassionate patient care i.e. doing something to get rid of patient's suffering	Share the feeling of patient's suffering (empathy)	Kindness
Ethics	Avoid any conflict of interest i.e., financial gain, sexual advantage, or other private purpose	Respect patient confidentiality and privacy	Adhere to doctor's religious and moral values
Medical competence	A holistic approach to patients i.e., treat the patient as a whole (mind, body, spirit, social)	Excellent knowledge and procedural skills	Able to treat patients in various situations with limited resources, i.e., mass casualty, disaster
Mindfulness and Self-reflectiveness	Responsive to feedback	Try new behaviors to promote patient care	Situational awareness i.e., able to detect and respond to any emergent situation in the ER
Appearance	Wearing a white coat	Professional attire i.e., shirt and tie, dress, closed-toe shoes	Appropriate grooming i.e., hairdressing and nail care
Information management	Complete medical records on time	Document charts accurately	Gather data efficiently with limited time and resources

Note: The cards were divided into 13 elements of emergency physician professionalism (EPP). Each element consisted of 3 cards describing unique behaviors related to EPP.
